# Triggers of viral encephalitis: a brain teaser to be solved!

**DOI:** 10.1186/s13613-025-01451-1

**Published:** 2025-03-18

**Authors:** Romain Sonneville, Annie Lannuzel, Cyrille Mathieu, Daniel Gonzalez-Dunia, Raphael Gaudin

**Affiliations:** 1 Université Paris Cité, INSERM U1137, IAME, Médecine intensive réanimation, AP-HP. Nord, Hôpital Bichat Claude Bernard, Paris, France; 2 Faculté de Médecine de l’Université des Antilles, Centre Hospitalier Universitaire de la Guadeloupe, Centre d’Investigation Clinique (CIC), Paris Brain Institute-ICM, CNRS, Hôpital de la Pitié Salpêtrière, Sorbonne Université, 97159 Pointe-à-Pitre, Inserm, Paris, 1424, 75013 France; 3 NeuroInvasion TROpism and VIRal Encephalitis Team, CIRI, Centre International de Recherche en Infectiologie, Univ Lyon, CNRS, UMR5308, ENS de Lyon, Inserm, U1111, Université Claude Bernard Lyon 1, 21 Avenue Tony Garnier, Lyon, F-69007 France; 4 Infinity, INSERM, CNRS, Université de Toulouse, Toulouse, France; 5 Institut de Recherche en Infectiologie de Montpellier (IRIM), Univ Montpellier, CNRS, 1919 route de Mende, Montpellier, 34293 France

Editorial

Although viral encephalitis is an uncommon cause of admission to hospital, its morbidity and mortality burden is disproportionately high. In a large prospective study on all-cause meningitis and encephalitis requiring care in the intensive care unit (ICU), viral encephalitis accounted for 17.1% of cases [1]. In this subset of patients, mortality was 17.2% and more than one-third of survivors were left with moderate to severe disability 90 days after admission. Survivors of infectious encephalitis often experience heavy functional and cognitive sequelae long after the infection resolves [2] and their burden may be even higher if the patients experienced prolonged ICU stay.

Over the years, a large panel of viruses associated with encephalitis have been identified; however, in more than half of the cases, the causative infectious agent remains unidentified and its nature varies depending on the geographical location [3, 4]. The reasons why a viral infection causes encephalitis remain poorly understood. Dysregulated immunity, comorbidities, and the genetic and epigenetic characteristics of patients are key factors that modulate this probability. Our own immune system is one of the main drivers responsible for viral encephalitis and its long-term functional outcomes, because it plays a critical role in disease development. While an immunosuppressed state influences CNS susceptibility to certain viruses, such as in case of measles inclusion bodies encephalitis (MIBE) or JC virus-induced progressive multifocal leukoencephalopathy (PML), an overwhelming host immune response to a neurotropic infection leads to profound neural damage. Indeed, pronounced neuroinflammation is associated to leukocyte infiltration into the CNS and the secretion of pro-inflammatory cytokines such as IL-1, IL-6, and TNFα. Along with antiviral treatment for selected etiologies, such as intravenous acyclovir for Herpes Simplex Virus (HSV) and Varicella Zoster Virus (VZV) encephalitis, anti-inflammatory treatments could also help in the management of viral encephalitis. Indeed, corticosteroids are already prescribed in about 40% of adult patients with suspected or confirmed encephalitis, but their benefit has yet to be demonstrated [5]. The DexEnceph multicenter randomized clinical trial, investigating the effects of adjunctive dexamethasone on outcome of HSV encephalitis will provide important information for delineating the use of steroids in this setting [6]. Other interesting immunomodulatory therapies for viral encephalitis include intravenous immunoglobulins, tocilizumab (an anti-IL-6 humanized monoclonal antibody), and anakinra (an IL-1 receptor antagonist).

The origin of neuroinflammation is thought to come from uncontrolled viral replication in the cells of the CNS, which would produce trigger warnings and initiate the immune response. This general concept, however, does not apply similarly for all viruses and one should make the distinction between three main types of viral neuroinfections: the brain-resident viruses, the hemorrhagic viruses (such as Ebola virus, Crimean-Congo hemorrhagic fever virus, Lassa virus, or in some cases Dengue virus), and the ones that do not fall into the two previous categories. For brain-resident viruses (mainly *herpesviridae*), an uncontrolled virus replication– due to immunodepression for instance– is the most likely cause of initial neuroinflammation, but it is not sufficient to fully explain the causes of bolting immunity in the brain. In the case of hemorrhagic fever viruses, infection of the endothelial cells composing the blood-brain barrier (BBB) is a likely cause of the initiation of neurological disorders and encephalitis, resulting in a permeable BBB that allows access of circulating virions by diffusion, as well as immune infiltration. Finally, in the third case, encephalitis is often the result of initial “stealthy” neuroinvasion, but can also originate from indirect peripheral infection only, or even after the infection has been cleared (as in acute post-infectious measles encephalitis [7]). In the former case, the molecular mechanisms by which virions (such as in the case of some arboviruses) can breach through an intact BBB to disseminate into the CNS remain obscure. Indeed, virus crossing of the BBB may be a turning point during a viral infection, which would tilt the balance from mild peripheral symptoms to life-threatening encephalopathy. Hence, therapeutic strategies aiming at preserving the BBB integrity have been proposed as a strategy to fight against viral neuroinfection [8]. However, such approaches need greater knowledge of the mechanisms involved in BBB maintenance to be able to safely translate it in clinical settings.

Intensivists are the first in line to treat viral encephalitis, and building more direct bridges between critical care doctors and researchers in neurobiology and virology is highly needed. Hence, to foster research on virus-induced neurological disorders, the French research agency INSERM created in 2024 the French “Virus & Brain” network. The goal of this network is to initiate discussions between researchers and clinicians treating infectious encephalitis whether from intensive care units, infectious diseases, or neurology departments. To this end, workshops and round tables have been organized in 2023 and 2024 in order to draw up the national landscape in the field, and we will continue with the organization of annual meetings at the national level, and an international conference planned for 2026. Furthermore, the network will promote the field by sponsoring young researchers and students to present their work at meetings. Together, the “Virus & Brain” network aims to federate the community to develop innovative lines of research and build original proposals based on interdisciplinary consortia united around a common goal. The main identified research themes include: the impact of viral neuroinfection, focusing on viral encephalitis; virus-induced neurocognitive disorders; and the link between exposome and neuroinfections.

The recent rise of biotechnologies, such as physiologically relevant in vitro culture models (organoids, organotypic cultures, etc.) and powerful omics approaches (transcriptomics, proteomics, single cell, spatial,…), must ultimately benefit the patients. We need to develop new therapeutic strategies to arm intensivists with alternative options to reduce the burden of viral encephalitis. This can be achieved by better linking the molecular information obtained in the lab to the symptomatic outcomes of patients admitted in ICU. By bridging the gap between the lab and the hospital, the French “Virus & Brain” network will actively promote bench-to-bedside research, potentially leading to seminal discoveries, promoting ambitious interdisciplinary projects and speeding up the translation of mechanistic observation in-a-dish into effective therapeutic strategies for patients.

## Data Availability

Not applicable.
